# Impact of IL-4/IL-13 Blockade with Dupilumab on the Microbiome in Type 2 Inflammatory Diseases: A Systematic Review

**DOI:** 10.1007/s11882-026-01281-6

**Published:** 2026-05-16

**Authors:** Pier-Valerio Mari, Lorenzo Carriera, Angela Saviano, Alberto Ricci, Angelo Coppola, Simone Ielo, Roberto Lipsi, Meridiana Dodaj, Francesco Gennari, Mario Gullà, Davide Stivalini, Maria Gabriella Pellegrino, Alessio Migneco, Eugenio De Corso, Veronica Ojetti

**Affiliations:** 1 Internal Medicine, San Carlo di Nancy Hospital, Rome, 00165 Italy; 2 Facoltà di Medicina e Chirurgia, Università Cattolica del Sacro Cuore, Largo F. Vito 1, Roma, 00168 Italy; 3 Department of Pulmonology and Sub-Intensive Respiratory Unit, Santa Maria della Misericordia Hospital, Piazzale Giorgio Menghini 3, Perugia, 06156 Italy; 4 Emergency Department, Fondazione Policlinico Universitario A. Gemelli IRCCS, Rome, 00168 Italy; 5 Department of Clinical and Molecular Medicine, Division of Pneumology, Sapienza University of Rome, AOU Sant’Andrea, Rome, 00189 Italy; 6 UOC Pneumologia, Ospedale San Filippo Neri-ASL Roma 1, Rome, 00135 Italy; 7 UniCamillus International Medical University of Rome, Rome, 00131 Italy; 8 Pulmonology and Respiratory Intensive Care Unit, Ospedale San Donato, USL Toscana Sud-Est, Arezzo, 52100 Italy; 9 Department of Medicine and Surgery, Section of Otorhinolaryngology, Santa Maria della Misericordia Hospital, Perugia, 06156 Italy

**Keywords:** Dupilumab, Type 2 inflammation, Microbiome, Microbiota, Gut-lung axis

## Abstract

**Purpose of Review:**

To systematically review current evidence on microbiota changes associated with dupilumab treatment across different anatomical sites in type 2 inflammatory diseases.

**Recent Findings:**

Fifteen studies were included, comprising two randomized trials and thirteen observational studies, mostly in atopic dermatitis, with fewer data in chronic rhinosinusitis with nasal polyps and NSAID-exacerbated respiratory disease. The skin was the most frequently investigated site, followed by the sinonasal tract and gut. Across skin studies, dupilumab was consistently associated with reduced *Staphylococcus aureus*, increased microbial diversity, and enrichment of commensal taxa. Sinonasal studies suggested shifts toward more eubiotic microbial communities. Gut evidence was limited, although one study suggested modulation of tryptophan metabolism-related pathways.

**Summary:**

Dupilumab appears to exert compartment-specific and disease-dependent effects on the microbiome. The strongest evidence concerns the skin and sinonasal compartments, whereas gut microbiota changes remain poorly defined. Further prospective studies are needed to assess microbiota signatures as potential biomarkers of response.

## Introduction

Type 2 (T2) inflammation represents a pathogenetic mechanism underlying several chronic diseases, including atopic dermatitis (AD), chronic rhinosinusitis with nasal polyps (CRSwNP), asthma, eosinophilic esophagitis (EoE), chronic spontaneous urticaria (CSU) and eosinophilic chronic obstructive pulmonary disease (COPD) [1–3]. T2-inflammation is driven by T helper type 2 (Th2) cells and type 2 innate lymphoid cells (ILC2) and mediated by type 2 cytokines, such as interleukin (IL)−4, IL-5, and IL-13, each with different roles in the inflammatory cascade. IL-4 drives IgE production and B-cell class switching, IL-13 causes mucus production, airway remodeling and bronchial hyperresponsiveness, and IL-5 is responsible for eosinophil differentiation, activation and survival [4]. Dupilumab, a fully human monoclonal antibody directed against the IL-4 receptor alpha subunit, simultaneously blocks both IL-4 and IL-13 signaling and has been approved for moderate-to-severe AD, CRSwNP, moderate-to-severe asthma with T2 inflammation, and eosinophilic esophagitis, with pivotal trials also demonstrating efficacy in eosinophilic COPD [5–7]. The human microbiome has emerged as a key player in immune homeostasis regulation. Dysbiosis has been consistently associated with T2 inflammatory disease pathogenesis [8, 9]. In the skin, increased *Staphylococcus aureus* colonization and reduced bacterial diversity are hallmarks of AD [9]. In the upper airways, CRSwNP patients exhibit a distinct nasal microbiota with enrichment in *Staphylococcus* and depletion of commensal genera such as *Corynebacterium* and *Dolosigranulum* [10]. In the lower airways, airway microbial composition has been linked to asthma severity and exacerbation frequency [11, 12]. In the gut, alterations in the *Firmicutes/Bacteroidetes* ratio and reduced short-chain fatty acid (SCFA) production have been implicated in systemic immune dysregulation through the gut-lung axis [13, 14]. The gut-lung axis represents a bidirectional communication pathway through which intestinal microbiota influences respiratory immunity and vice versa [15]. Microbial metabolites, including SCFAs, tryptophan derivatives, and secondary bile acids, can influence immune regulation at distant mucosal sites by modulating dendritic cell activity, regulatory T-cell differentiation, and epithelial barrier integrity [16]. Recently the role of gut microbiota dysbiosis and its metabolic consequences in immune mediated diseases has been investigated, highlighting the importance of SCFA production [17], tryptophan metabolism [18, 19], smoking-induced gut dysbiosis [20], and intestinal permeability as drivers of systemic inflammation [21, 22]. Collectively, these findings provide a strong rationale for investigating whether pharmacological modulation of T2 inflammation may indirectly reshape the microbiome. While dupilumab clinical efficacy is well established, its impact on the host microbiome has received limited attention. Recent data point to several mechanisms through which IL-4/IL-13 blockade may reshape microbial communities: restoration of epithelial barrier function, modulation of antimicrobial peptide production, reduction of IgE-mediated mast cell activation, and alteration of the local inflammatory milieu [23, 24]. Moreover, IL-4 and IL-13 directly regulate goblet cell differentiation, mucin production, and epithelial tight junction expression, all determinants of microbial niche selection [25, 26]. To date, no systematic review has comprehensively synthesized evidence on dupilumab-associated microbiota changes across different anatomical compartments and disease contexts. The aim of this systematic review is to synthesize available evidence on dupilumab impact on microbiota composition across different anatomical sites.

## Materials and Methods

### Study Design and Protocol Registration

This systematic review was conducted in accordance with the Preferred Reporting Items for Systematic Reviews and Meta-Analyses (PRISMA) 2020 statement [27]. The protocol was registered in the International Prospective Register of Systematic Reviews (PROSPERO) before the study was conducted (registration number: CRD420261331758).

### Eligibility Criteria

A PICO specialized framework was used to define the search strategy considering: P (Population): Patients with T2 inflammatory diseases, including AD, CRSwNP, NSAID-exacerbated respiratory disease (N-ERD), asthma, COPD, EoE, CSU. I/E (Intervention/Exposure): Treatment with dupilumab. C (Comparison): Pre-treatment status, placebo, other treatments, healthy controls. O (Outcomes): Changes in microbiota composition, diversity, or specific taxa identification at any anatomical site (skin, nasal/sinonasal, gut, ocular, airway/bronchial).

This review aimed to answer the following focused research question: Does treatment with dupilumab influence the composition or diversity of the microbiota across different anatomical sites in patients with type 2 inflammatory diseases?

### Search Strategy

A systematic literature search was performed on the online databases MEDLINE (PubMed), Scopus, ClinicalTrials.gov, Cochrane Library, Google Scholar from inception to March 2026, and was followed by manual literature searches in the reference lists of the included articles to identify potential additional articles about this topic. The research string was as follows: *(dupilumab) AND (microbiome OR microbiota)*.

### Study Selection

We included randomized controlled trials, prospective and retrospective observational studies, case series that met the following inclusion criteria: 1) patients with a known history of T2 inflammatory disease receiving dupilumab or another biologic agent, 2), outcomes describing microbiota data, 3) articles written in English. Exclusion criteria included animal studies, in vitro studies, conference abstracts without full-text data, and studies without microbiota-specific endpoints.

### Data Extraction

Two researchers (PVM and LC) independently searched the data, selected and extracted them. If the extracted data from both researchers did not match, the original text was reviewed by another researcher (AC) to reach a consensus. Extracted data included the following items: author/year, study design, sample size, type of T2 inflammatory disease, intervention and comparator, anatomical site of microbiota sampling, microbiota assessment methodology (16 S rRNA sequencing, culture-based, metagenomics, metabolomics), primary microbiota outcomes (alpha diversity, beta diversity, specific taxa changes), associated clinical outcomes, and follow-up duration.

### Evaluating the Risk of Bias

Risk-of-bias assessment was performed independently by two reviewers (PVM and LC), with disagreements resolved by consensus or a third reviewer (AC). The risk of bias was assessed using the Cochrane Risk of Bias 2 (RoB 2) tool for randomized controlled trials [28], while for non-randomized studies it was assessed using the Risk Of Bias In Non-randomized Studies of Interventions (ROBINS-I) tool [29] and the Joanna Briggs Institute’s (JBI) Critical Appraisal Checklist for Case Series [30].

### Data Synthesis

Given the heterogeneity of outcomes assessed across studies, data from original papers were extracted and reported via qualitative synthesis.

## Results

### Study Selection

**Fig. 1:**
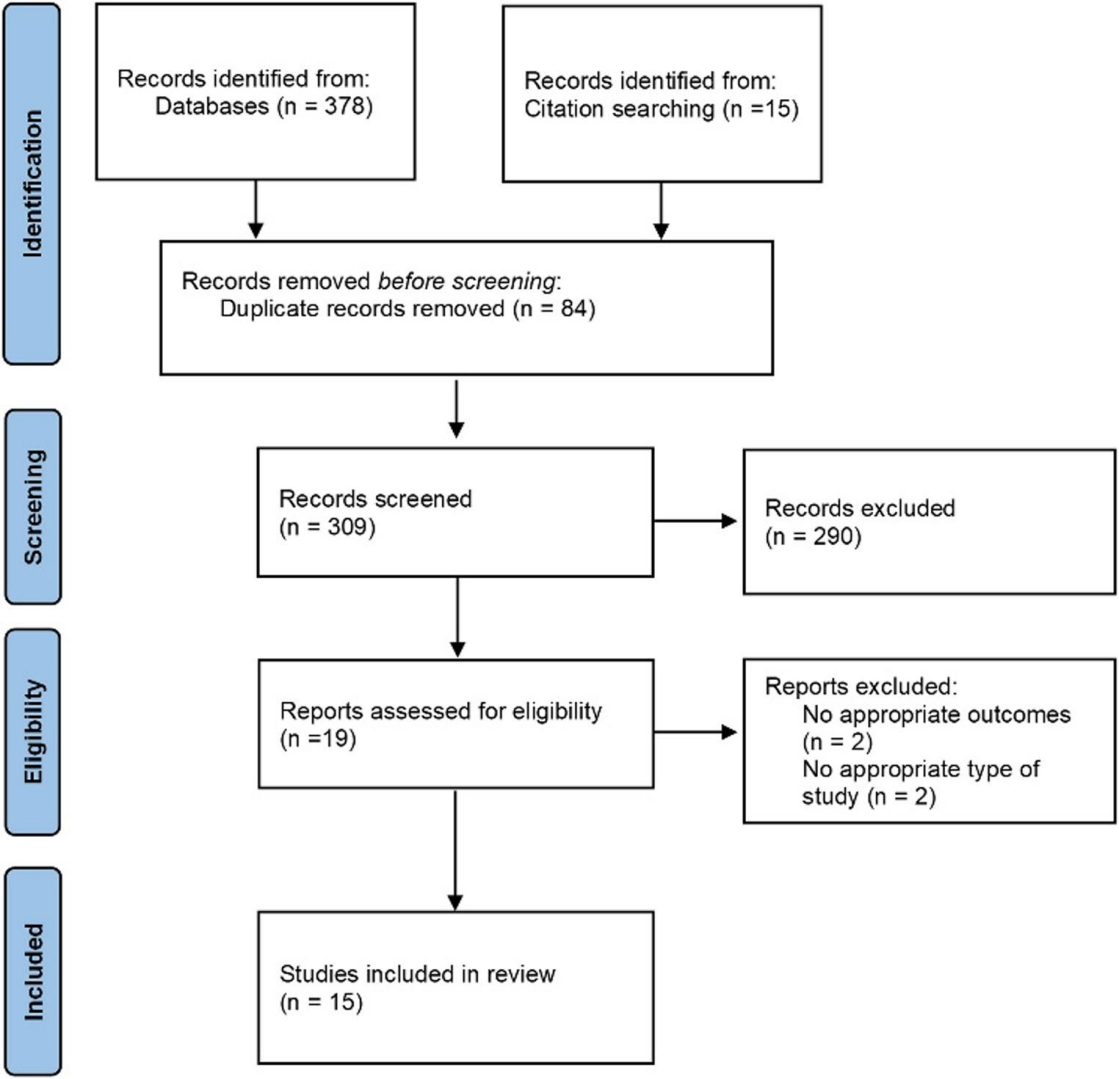
PRISMA flow diagram illustrating the study selection process

The initial literature search generated 378 potentially eligible articles from the aforementioned databases, plus 15 record identified additionally by manual search. A total of 84 duplicates were identified and removed. After excluding 290 articles, 15 articles were included in this review according to the prespecified inclusion and exclusion criteria. A flow chart showing the study selection is presented in Fig. 1.

### Characteristics of the Included Studies

A total of 15 studies were included in the review, comprising two randomized placebo-controlled trials and thirteen observational studies [31–45]. The studies were published between 2020 and 2026 and investigated the effects of dupilumab therapy on microbial communities across different anatomical sites in patients with type-2 inflammatory diseases. The majority of studies focused on AD (*n* = 11), while four studies investigated patients with CRSwNP or N-ERD. Sample sizes ranged from 19 to 130 patients receiving dupilumab therapy. Most investigations evaluated skin microbiota (*n* = 9); three studies examined the nasal microbiota; one study assessed both skin and nasal microbiota; one study assessed both nose and gut microbiota and one study explored only gut microbiota. Microbial composition was primarily assessed using 16 S rRNA gene sequencing, often combined with quantitative PCR, while some studies employed culture-based methods, next-generation sequencing, or fungal ITS/28S rRNA sequencing to characterize bacterial and fungal communities. Comparator groups varied across studies. The randomized trials used placebo controls, while observational studies compared dupilumab-treated patients with healthy controls, untreated disease cohorts, or alternative treatments, including surgery, Janus kinase inhibitors, cyclosporine A, and upadacitinib. Several studies were single-arm observational cohorts without a comparator group. Across studies, dupilumab treatment was consistently associated with modulation of microbial composition and diversity, particularly in the skin microbiome of patients with atopic dermatitis. Many studies reported increased microbial α-diversity and a reduction in the relative abundance of *Staphylococcus aureus* following treatment, accompanied by increases in commensal taxa such as *Staphylococcus epidermidis*, *Staphylococcus hominis*, and *Cutibacterium acnes*. Similar shifts toward a more balanced microbial community were observed in nasal microbiota in patients with CRSwNP, with increased representation of genera such as *Corynebacterium*, *Dolosigranulum*, and *Lawsonella*. In contrast, studies examining the gut microbiota reported more modest or limited changes during dupilumab therapy. Follow-up durations ranged from 4 weeks to 6 months, with most studies reporting microbiome changes emerging within the first weeks of treatment and persisting throughout the observation period. A detailed summary of study characteristics is provided in Table 1.

**Table 1:** Studies Investigating the Effect of Dupilumab on Microbiota

Author (Year)	Study Design	Sample Size	Disease	Anatomical site	Methods of assessment	Comparator	Outcomes	Follow-up
Callewaert (2020) [31]	Randomized placebo controlled trial	54 patients Intervention *n* = 27	AD	Skin	16 S rRNA Gene Sequencing, qPCR	Placebo *n* = 27	Treatment with dupilumab significantly changed the composition of the microbiome and increased microbial diversity while reducing the abundance of *S. aureus*. Increased diversity was observed as early as week 4	16 weeks
Simpson (2023) [32]	Randomized placebo-controlled trial	72 patients Intervention *n* = 45	AD	Skin	16 s rRNA gene sequencing	Placebo *n* = 26	Dupilumab treatment increased the Shannon microbial α-diversity in lesional (but not nonlesional) skin as early as 3 days, with near maximal effect by day 28. Progressive reduction in the relative abundance of *S aureus* (both lesional and nonlesional skin) and increases of *S epidermidis*, *Cutibacterium* acne, *S hominis* and *Micrococcus luteus*.	16 weeks
Ryser (2025) [33]	Prospective case–control study	48 patients Intervention *n* = 27	CRSwNP	Nose and gut	16 s rRNA gene amplicon sequencing	CRSwNP untreated patients (*n* = 10), healthy controls (*n* = 11)	Dupilumab treatment was associated with increased relative abundances in the nose of genera such as *Lawsonella*, *Corynebacterium*, and *Dolosigranulum*. The abundance of *Lactobacillus spp*. decreased. There were no changes in gastrointestinal microbiota during dupilumab treatment.	180 days
Maniaci (2024) [34]	Prospective observational study	44 patients Intervention *n* = 22	CRSwNP	Nose	Culture + PCR	Surgery *n* = 22	In the dupilumab group, *Staphylococcus epidermidis* prevalence increased while *Pseudomonas aeruginosa* was eradicated. Dupilumab stabilised *Staphylococcus aureus* prevalence compared to surgery group	6 months
Aluisio (2026) [35]	Prospective observational study	44 patients Intervention *n* = 22 Surgery *n* = 22	CRSwNP	Nose	Culture	None	At baseline, the most prevalent bacteria were *Staphylococcus aureus*,* Stapylococcus epidermidis* and *Pseudomonas aeruginosa*. After 6 months, *S. aureus* and *S. epidermidis* significantly increased while *P. aeruginosa* decreased.	6 months
Bartosik (2025) [36]	Prospective observational study	28 patients Intervention *n* = 28	N-ERD	Nose	16 s rRNA gene amplicon sequencing	None	No major changes in microbiome diversity or composition observed	24 weeks
Olesen (2021) [37]	Prospective observational study	27 patients Intervention *n* = 27	AD	Skin and nose	16 s rRNA and tuf gene sequencing.	None	Skin: increases in *S. epidermidis* and *S. hominis*, decrease in *S. aureus*. Nose: Shannon diversity increased. Increase in *Rothia*, *Corynebacterium*, *Haemophilus*, *Veillonella*	16 weeks
Yang (2024) [38]	Prospective observational cohort study	92 patients Intervention *n* = 27	AD	Gut	16 s rRNA gene sequencing	Healthy controls *n* = 48; AD untreated patients *n* = 27	After dupilumab treatment, increased colonization of *Bifidobacterium*, *Ruminococcus gnavus*, and *Coprococcus*. Up-regulated expression of genes involved in the indole pathway of tryptophan metabolism	16 weeks
Pažur (2023) [39]	Prospective observational study	50 patients Intervention *n* = 50	AD	Skin	NGS + qPCR	None	Ratio of *S. aureus* to *S. epidermidis* decreased significantly after dupilumab therapy. A similar finding was seen in the ratio of *S. aureus* to *C. acnes*. The ratio of *C. acnes* to *S. epidermidis* remained constant during the observed period	6 months
Hartmann (2023) [40]	Prospective observational study	157 patients Intervention *n* = 130 Cyclosporine *n* = 27	AD	Skin	16 s rRNA gene sequencing	Healthy controls *n* = 258	Dupilumab therapy shifted bacterial community toward the pattern seen in healthy controls. The relative abundance of *staphylococci* and in particular *S.aureus* significantly decreased on both lesional and non lesional skin, whereas the abundance of *staphylococcus hominis* increased.	3 months
Umemoto (2024) [41]	Prospective observational study	30 patients Intervention *n* = 30	AD	Skin	Fungal 28 S rRNA Gene Sequencing, Bacterial 16 S rRNA Gene Sequencing, qPCR	Healthy controls *n* = 10	Decrease in *Malassezia* colonization. Decrease of *M restricta* and *M. globosa*, reduction of *Staphylococcus aureus* and increase in the microbial diversity.	12-weeks
Veronese (2026) [42]	Prospective observational study	23 patients Intervention *n* = 12	AD	Skin	Culture and PCR	Upadacitinib *n* = 11	Both dupilumab and upadacitinib effectively reduced disease severity and *S. aureus* abundance across lesional and nonlesional sites, with upadacitinib showing faster clinical improvement	16 weeks
Thomova (2026) [43]	Prospective observational study	60 patients Intervention *n* = 21	AD	Skin	16 S rRNA Gene Sequencing and qPCR	Jak inhibitors (*n* = 8), cyclosporine A (*n* = 14) and topical corticosteroids (*n* = 17)	Dupilumab resulted in complete depletion of *Staphylococcus aureus*. Although overall bacteriome alpha diversity remained unchanged, the ratio of *Staphylococcus* to *Corynebacterium* and *Cutibacterium* decreased significantly after Jak inhibitors as well as dupilumab treatment.	6 months
Li (2025) [44]	Prospective observational study	24 patients Intervention *n* = 11	AD	Skin	DNA sequencing	Healthy controls *n* = 15; upadacitinib *n* = 13	After 4 months of dupilumab treatment, the abundance of *Malassezia spp*. initially decreased but later returned to base line levels. In contrast, after treatment with upadacitinib, the relative abundance of *Malassezia spp*. was restored to levels similar to those of healthy individuals	16 weeks
Koike (2025) [45]	Prospective observational study	19 patients Intervention *n* = 13	AD	Skin	Fungal ITS1 deep sequencing, bioinformatic analysis, and taxonomic assignment	cyclosporine A (*n* = 6)	Treatment with dupilumab and cyclosporine was associated with shifts toward higher *Malassezia* abundance and modulation between *M. restricta* and *M. globosa.*	4–8 weeks

Legend of abbreviations: 16 S rRNA: 16 S ribosomal RNA; 28 S rRNA: 28 S ribosomal RNA; AD: atopic dermatitis; C. acnes: Cutibacterium acnes; CRSwNP: chronic rhinosinusitis with nasal polyps; DNA: deoxyribonucleic acid; ITS1: internal transcribed spacer 1; Lactobacillus spp.: Lactobacillus species; M. globosa: Malassezia globosa; M. restricta: Malassezia restricta; Malassezia spp.: Malassezia species; n: number of patients; N-ERD: NSAID-exacerbated respiratory disease; NGS: next-generation sequencing; NSAID: non-steroidal anti-inflammatory drug; P. aeruginosa: Pseudomonas aeruginosa; PCR: polymerase chain reaction; qPCR: quantitative polymerase chain reaction; S. aureus: Staphylococcus aureus; S. epidermidis: Staphylococcus epidermidis; S. hominis: Staphylococcus hominis; tuf gene: elongation factor Tu gene

### Risk of Bias Assessment

**Fig. 2:**
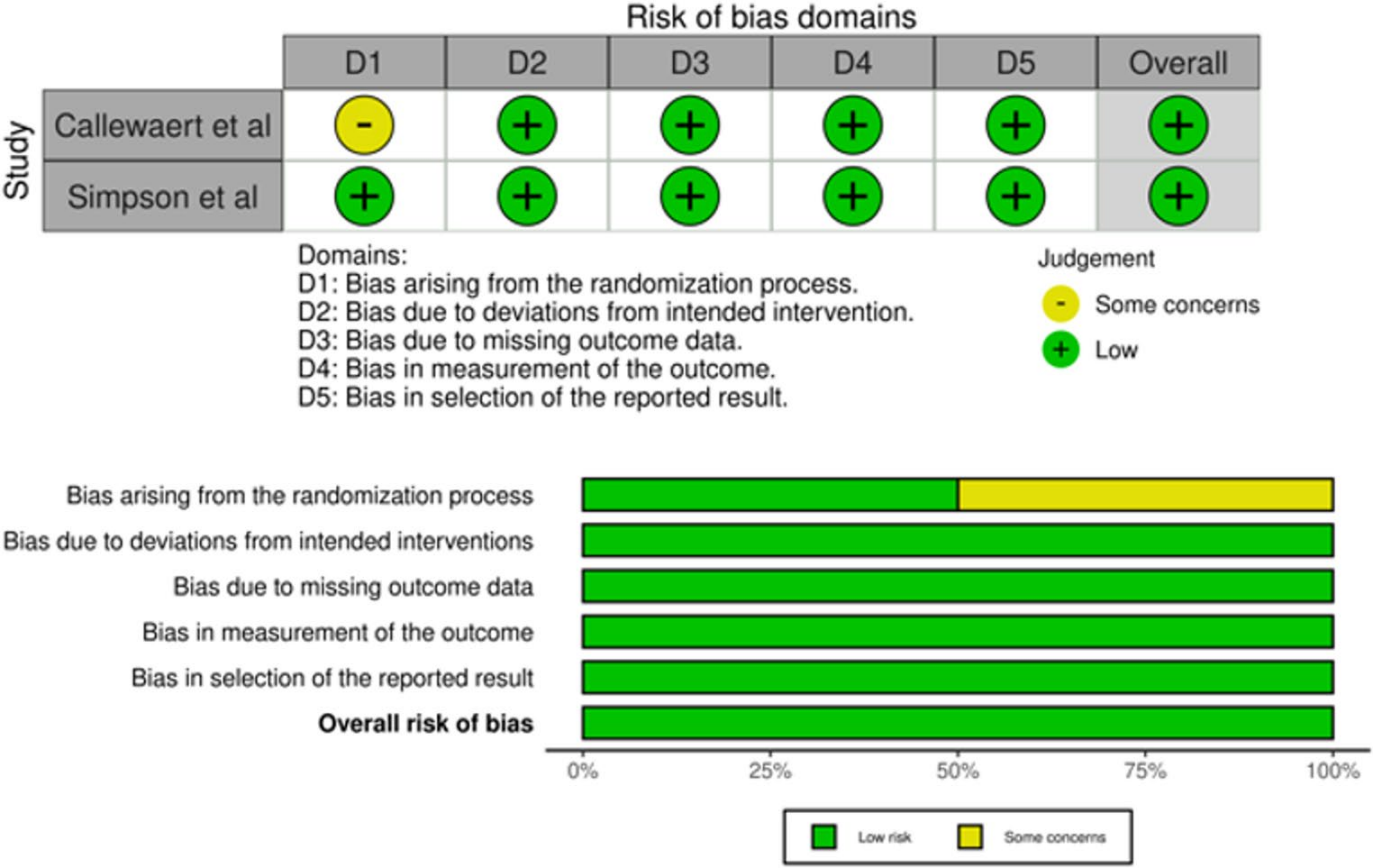
Risk of bias assessment with Cochrane Risk of Bias 2 (RoB 2), traffic and plot graph

**Fig. 3:**
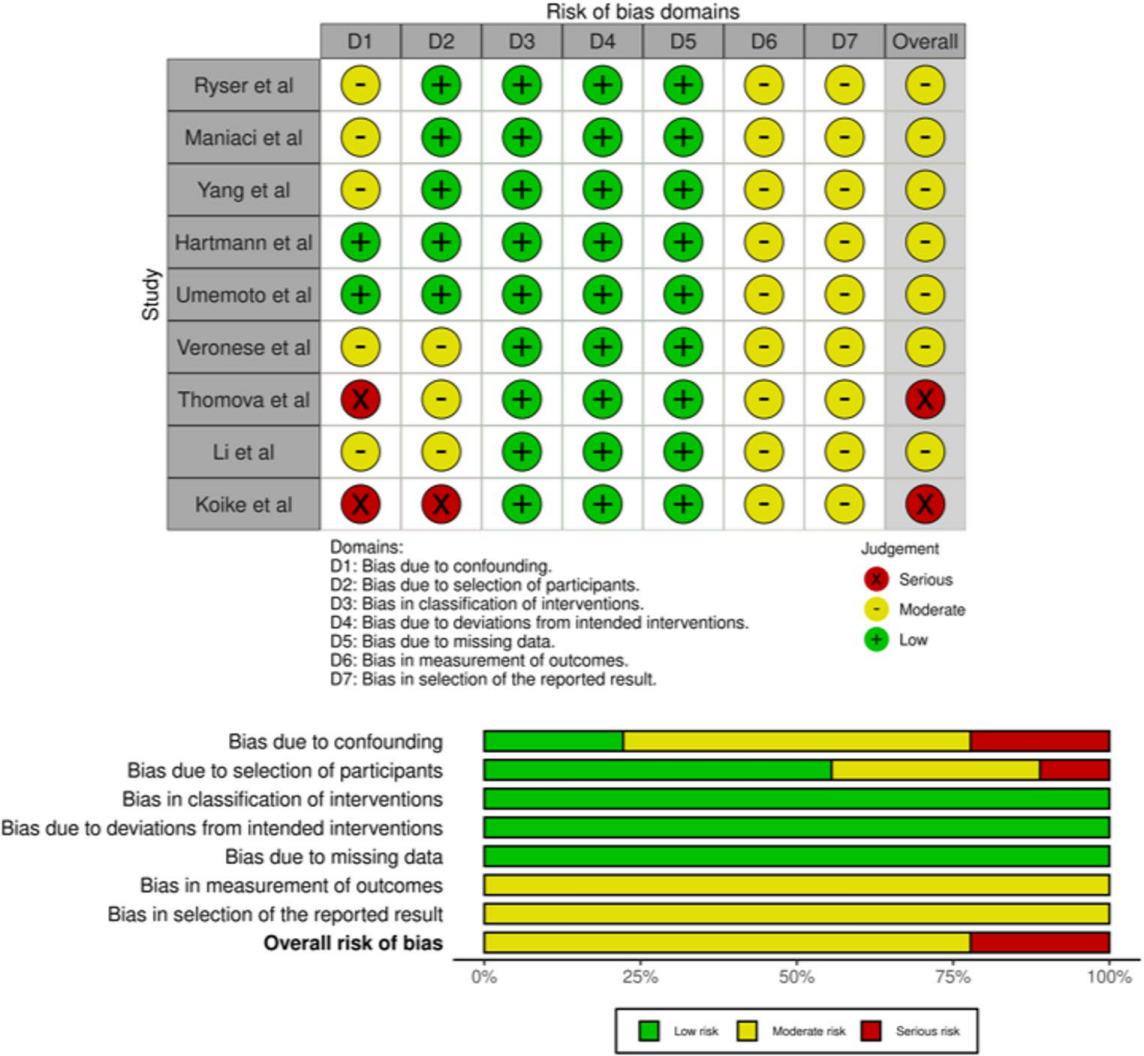
Risk of bias assessment with the Risk Of Bias In Non-randomized Studies of Interventions (ROBINS-I), traffic and plot graph

**Fig. 4:**
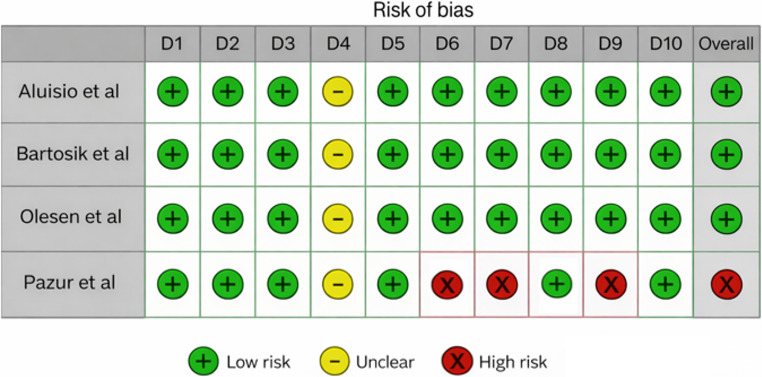
Risk of bias assessment with the Joanna Briggs Institute’s (JBI) Critical Appraisal Checklist for Case Series, traffic graph

The risk of bias was evaluated according to study design using the appropriate tools. Among the randomized studies assessed with the RoB 2, both were judged to be at low risk of bias. For non-randomized comparative studies evaluated with ROBINS-I, seven studies were rated as having a moderate risk of bias, and two as having a serious risk of bias. For observational studies without a comparator group, risk of bias was assessed using the Joanna Briggs Institute Critical Appraisal Checklist for Case Series, which showed generally acceptable quality despite methodological limitations, with three studies classified as low risk of bias and one study as having a serious risk of bias. Overall, most included studies demonstrated low to moderate risk of bias. The quality assessment results are reported for randomized studies (Fig. 2), non-randomized comparative studies (Fig. 3) and observational studies without a comparator group (Fig. 4).

## Discussion

The skin is the compartment most extensively investigated in relation to dupilumab-associated microbiome changes, particularly in patients with atopic dermatitis. Across the studies included in this review, a relatively consistent pattern emerged, suggesting that dupilumab treatment may be associated with a progressive shift of the cutaneous microbiota toward a more balanced microbial profile. This trend was characterized by a reduction in *Staphylococcus aureus* abundance, an increase in microbial diversity, and a relative enrichment of commensal taxa commonly associated with skin homeostasis. A central finding across studies was the reduction of *S. aureus*, a well-recognized hallmark of atopic dermatitis dysbiosis [46, 47]. In parallel with the reduction in *S. aureus*, several studies documented increases in taxa generally considered commensal or potentially beneficial in the context of skin health. These included *S. epidermidis*,* S. hominis*, and *C. acnes* [32, 37, 39, 40],. Their increased relative abundance may reflect restoration of a microbial environment less dominated by inflammatory dysbiosis. The temporal dynamics observed across studies are noteworthy. Some changes appeared rapidly, and increases in diversity and reductions in S. aureus were detectable within days to weeks of treatment initiation. At the same time, longer observational studies suggest that these changes may persist over several months, indicating that dupilumab-related modulation of the skin microbiota is not limited to a transient early effect. A smaller but relevant group of studies also examined the cutaneous mycobiome. Findings suggest that fungal responses may be more variable and potentially more sensitive to timing than bacterial responses. Therefore, while the bacterial component of the skin microbiota appears to show a relatively coherent pattern under dupilumab, current evidence on the cutaneous mycobiome remains less consistent. Some limitations hinder generalizability. The included studies differed substantially in microbiome assessment methods, sampling strategies, comparator groups, and follow-up duration. Therefore, the extent to which these changes are causally linked to immune modulation and barrier restoration remains to be clarified. The nasal and sinonasal microbiota represent a key component of the mucosal ecosystem involved in upper airway homeostasis and chronic inflammatory diseases such as CRSwNP and N-ERD. In these conditions, microbial dysbiosis has been described, frequently characterized by reduced bacterial diversity and enrichment of opportunistic pathogens, including *Staphylococcus aureus*. Evidence from observational studies indicates that dupilumab therapy may promote shifts in the nasal microbiota toward taxa more commonly observed in healthy individuals. In particular, increased relative abundances of commensal genera such as *Corynebacterium*, *Dolosigranulum*, and *Lawsonella* have been reported following treatment [33]. These taxa are often considered components of a stable and health-associated nasal microbiome and have been proposed to play a role in maintaining epithelial barrier integrity and competitive exclusion of pathogenic bacteria. At the same time, reductions in potentially pathogenic organisms, including *Pseudomonas aeruginosa* or alterations in the prevalence of *Staphylococcus* species, have been described [34]. Although the magnitude and consistency of these changes vary across studies, the available data suggest that IL-4/IL-13 blockade may contribute to a gradual rebalancing of sinonasal microbial communities. In CRSwNP and related diseases, type-2 cytokines contribute to epithelial barrier dysfunction, mucus hypersecretion, and altered antimicrobial peptide expression. These alterations may create a permissive environment for microbial dysbiosis. Consequently, the improvement of epithelial barrier function and reduction of local inflammation following dupilumab therapy could indirectly reshape the microbial niche, allowing the expansion of commensal taxa and reducing the dominance of opportunistic pathogens. Compared with the skin and sinonasal compartments, the gut microbiota has been investigated much less frequently in patients receiving dupilumab. Only two studies included in this review explored this compartment, and their findings suggest a less consistent and less pronounced effect than that observed in the skin [33, 38]. The most relevant signal comes from the study by Yang et al. [38], which reported that dupilumab treatment was associated with increased colonization by taxa such as *Bifidobacterium*, *Ruminococcus gnavus*, and *Coprococcus*. Bacterial functional analysis revealed upregulated pathway of tryptophan metabolism after dupilumab, generating metabolites such as indole-3-propionic acid and indole-3-aldehyde, which are ligands for the aryl hydrocarbon receptor (AhR) promoting epithelial barrier integrity and regulatory T-cell differentiation.

**Fig. 5:**
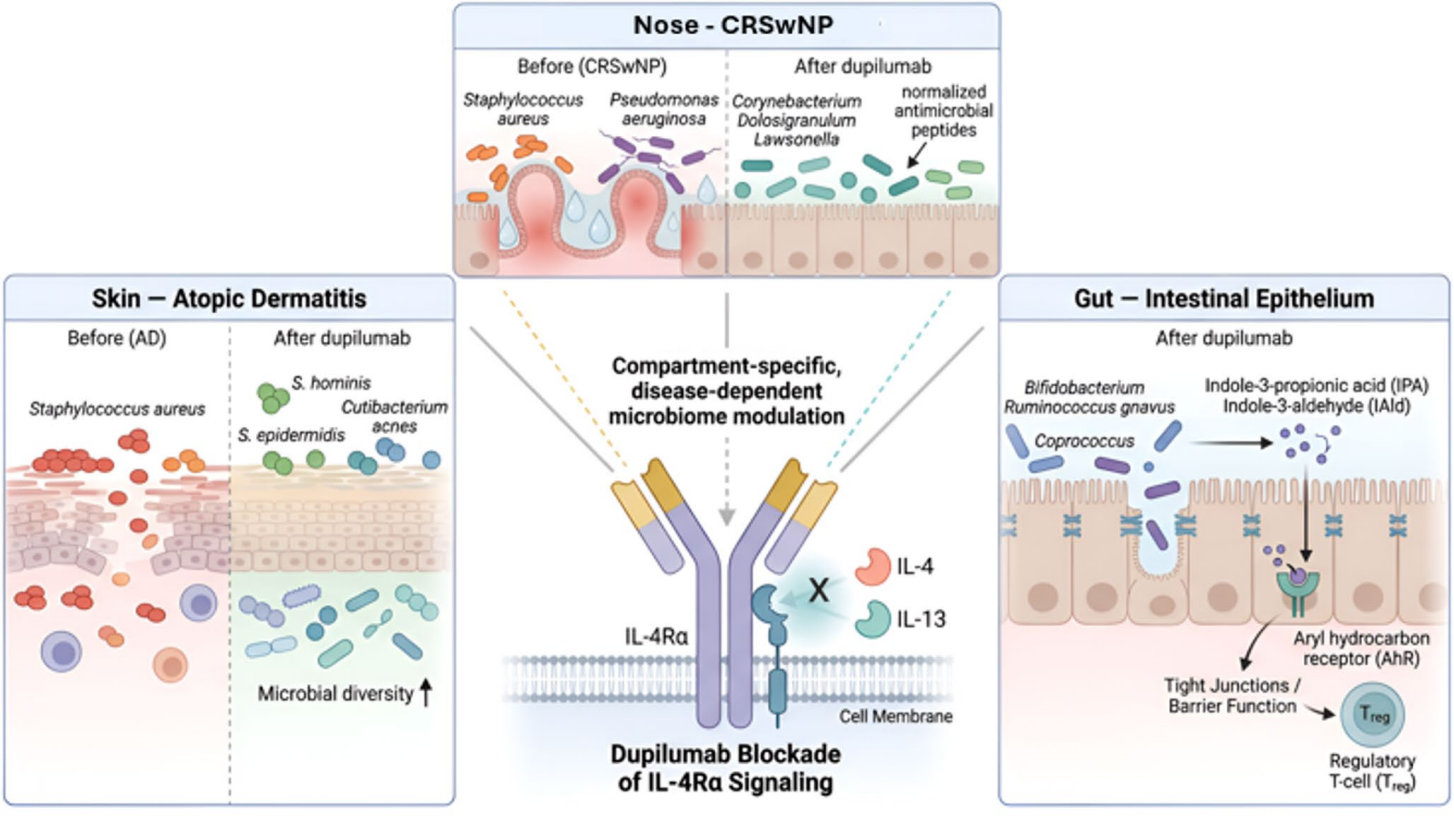
Compartment-specific microbiome changes associated with dupilumab. Dupilumab blocks IL-4Rα signaling, inhibiting IL-4 and IL-13 pathways and promoting site-specific microbiome modulation. In atopic dermatitis, treatment is associated with reduced *Staphylococcus aureus*, increased commensal skin taxa, and greater microbial diversity. In CRSwNP, it is linked to a shift from dysbiosis toward a more balanced nasal microbiome. In the gut, dupilumab-associated changes include enrichment of beneficial taxa and microbial metabolites, with potential effects on epithelial barrier function and immune regulation

In contrast, Ryser et al. [33] did not detect relevant changes in the gut microbiota during dupilumab treatment for CRSwNP, despite observing shifts in the sinonasal compartment. This finding suggests that the microbiological impact of dupilumab may be more pronounced in tissues directly affected by type-2 inflammatory processes rather than reflecting a systemic microbiome shift. At present, the interpretation of gut microbiome findings remains necessarily cautious. The number of available studies is very small, sample sizes are limited, and the reported outcomes are heterogeneous. Moreover, the gut microbiota is shaped by a wide range of potential confounders, including diet, body mass index, antibiotic exposure, concomitant medications, and comorbidities, which were not uniformly controlled across studies. Notably, other anti-T2 biologics have shown limited or no detectable effects on microbial composition. In particular, anti-IL-5 therapies did not significantly alter airway microbiota despite effective eosinophil depletion [48, 49], while anti-IgE therapy has shown only preliminary and compartment-specific microbiome associations [50]. Overall, the available evidence suggests that dupilumab may exert compartment-specific and disease-dependent effects on the host microbiome, a dimension of its pharmacological activity that has received relatively limited attention. In particular, the observed microbiome changes are compatible with the hypothesis that IL-4/IL-13 blockade contributes to the restoration of epithelial barrier function and antimicrobial peptide balance, potentially creating conditions that favor recolonization by commensal microbial communities (Fig. 5).

### Study Limitations

This systematic review has several limitations. The evidence base is predominantly observational and derived from small cohorts, which constrains generalizability. Microbiota assessment methodologies varied considerably across studies, from culture-based techniques to 16 S rRNA gene sequencing and metagenomics, making direct quantitative comparisons unfeasible. Most studies reported short-term outcomes, leaving the long-term stability of dupilumab-associated microbiota changes undetermined. Potential confounders, including concomitant medications (antibiotics, intranasal corticosteroids, proton pump inhibitors), dietary habits, and geographic variability, were inconsistently controlled. A bias toward reporting positive findings may have influenced the apparent consistency of microbiome changes in the nasal and skin compartments. The lack of standardized microbiome endpoints across studies hampers comparability. Finally, the scarcity of data on the gut and airway compartments constitutes a major evidence gap that precludes definitive conclusions regarding the systemic microbiome effects of IL-4/IL-13 blockade.

## Conclusions

This systematic review suggests that dupilumab may exert compartment-specific and disease-dependent effects on the host microbiome. The available evidence indicates that IL-4/IL-13 blockade may be associated with shifts toward microbial communities more frequently observed in healthy skin and nasal environments, while limited data from the gut compartment suggest a possible modulation of pathways related to tryptophan metabolism. Comparisons with other anti-T2 biologics tentatively suggest that microbiome changes may not represent a universal feature of anti-eosinophilic therapies, but could be more closely linked to IL-4/IL-13 antagonism. A notable gap in the current literature is the lack of studies investigating the bronchial or pulmonary microbiome, which may be particularly relevant given the expanding clinical indications of dupilumab in eosinophilic COPD. In this context, the gut–lung axis represents a potentially important yet still insufficiently explored pathway that could help explain the broader biological effects of dupilumab across mucosal compartments. Prospective, multi-compartment studies incorporating standardized sampling and microbiological analysis are needed to determine whether microbiota composition can serve as a predictive biomarker of treatment response to dupilumab across T2 inflammatory diseases.

## Key References

- Fujimura KE, Lynch SV. Microbiota in allergy and asthma and the emerging relationship with the gut microbiome. Cell Host Microbe. 2015 May 13;17 [5]:592–602. doi:10.1016/j.chom.2015.04.007.
  - ◦This review is an important conceptual reference linking allergy, asthma, and the gut microbiome. It helps frame the broader biological rationale for microbiome involvement in type 2 inflammatory diseases and supports the relevance of exploring gut microbial pathways in dupilumab-treated patients.
- Hoggard M, Wagner Mackenzie B, Jain R, Taylor MW, Biswas K, Douglas RG. Chronic Rhinosinusitis and the Evolving Understanding of Microbial Ecology in Chronic Inflammatory Mucosal Disease. Clin Microbiol Rev. 2017 Jan;30 [1]:321–48. doi:10.1128/CMR.00060-16.
  - ◦This is a key background review on the microbial ecology of chronic rhinosinusitis. It provides the mechanistic and ecological framework needed to interpret sinonasal microbiota changes in inflammatory airway disease and helps contextualize microbiome shifts observed with biologic treatment.
- D’Avino P, Kim J, Li M, Gessner P, Westermann P, Pat Y, et al. Distinct Roles of IL-4, IL-13, and IL-22 in Human Skin Barrier Dysfunction and Atopic Dermatitis. Allergy. 2026 Feb;81 [2]:480–97. doi:10.1111/all.70060.
  - ◦This study is particularly relevant because it clarifies the distinct contribution of type 2 cytokines to skin barrier dysfunction in atopic dermatitis. It strengthens the biological plausibility that IL-4/IL-13 blockade may indirectly reshape the skin microbiome by restoring epithelial barrier integrity and reducing inflammatory dysbiosis.
- Ryser FS, Demeter T, Pijuan JB, Shambat SM, Brühlmann C, Mauthe T, et al. Dupilumab Treatment Is Associated With Clinical Improvement and a Shift Toward a Health-Associated Nasal Passage Microbiota in Diffuse Type 2 Chronic Rhinosinusitis. Allergy. 2025 Jun;80 [6]:1746–56. doi:10.1111/all.16600.
  - ◦This is a highly relevant study directly showing that dupilumab treatment is associated not only with clinical improvement but also with a shift toward a healthier nasal microbiota profile in type 2 chronic rhinosinusitis. It provides direct support for the idea that dupilumab may modulate microbiome composition in a compartment-specific manner.
- Maniaci A, Vertillo Aluisio G, Stefani S, Cocuzza S, Lechien JR, Radulesco T, et al. Differential Nasal Recolonization and Microbial Profiles in Chronic Rhinosinusitis With Nasal Polyps Patients After Endoscopic Sinus Surgery or Dupilumab Treatment: A Prospective Observational Study. Clinical Otolaryngology. 2025 Mar;50 [2]:262–70. doi:10.1111/coa.14246.
  - ◦This prospective observational study is important because it compares microbial profiles after different therapeutic strategies, endoscopic sinus surgery and dupilumab. It offers useful insight into how biologic therapy may influence nasal recolonization patterns differently from surgical intervention, helping to refine interpretation of microbiota changes in CRSwNP.

## Data Availability

All data analyzed are included in the article.
